# At the heart of inflammation: Unravelling cardiac resident macrophage biology

**DOI:** 10.1111/jcmm.70050

**Published:** 2024-09-02

**Authors:** Yingnan Liao, Liyuan Zhu

**Affiliations:** ^1^ Sichuan Provincial Key Laboratory for Human Disease Gene Study and the Center for Medical Genetics, Department of Laboratory Medicine, Sichuan Academy of Medical Sciences and Sichuan Provincial People's Hospital University of Electronic Science and Technology of China Chengdu China; ^2^ Research Unit for Blindness Prevention, Chinese Academy of Medical Sciences (2019RU026) Sichuan Academy of Medical Sciences and Sichuan Provincial People's Hospital Chengdu Sichuan China; ^3^ Center of Clinical Pharmacology, The Second Affiliated Hospital, School of Medicine Zhejiang University Hangzhou China

**Keywords:** cardiac repair, cardiac resident macrophages, cardiovascular diseases, CCR2, inflammation

## Abstract

Cardiovascular disease remains one of the leading causes of death globally. Recent advancements in sequencing technologies have led to the identification of a unique population of macrophages within the heart, termed cardiac resident macrophages (CRMs), which exhibit self‐renewal capabilities and play crucial roles in regulating cardiac homeostasis, inflammation, as well as injury and repair processes. This literature review aims to elucidate the origin and phenotypic characteristics of CRMs, comprehensively outline their contributions to cardiac homeostasis and further summarize their functional roles and molecular mechanisms implicated in the onset and progression of cardiovascular diseases. These insights are poised to pave the way for novel therapeutic strategies centred on targeted interventions based on the distinctive properties of resident macrophages.

## 1 INTRODUCTION

In 2012, chronic diseases accounted for 68% of global mortality, with cardiovascular disease emerging as the predominant cause. By 2019, the worldwide prevalence of cardiovascular conditions had reached 523 million individuals, leading to 18.6 million deaths.^1^ Over the past four decades, China has witnessed a concerning escalation in the rates of cardiovascular diseases, with ischemic heart disease now ranking as the second leading cause of mortality,^2^ even among younger demographics.^3^ Consequently, cardiovascular disease emerges as the foremost public health challenge in world.^4^

In cardiovascular diseases, chronic low‐grade inflammation is a persistent feature that contributes to both the onset and progression of the disease. Effective regulation of this inflammation has emerged as a promising strategy for intervention. Among the immune cells involved in cardiovascular inflammation, macrophages are the earliest to appear during development, are the most prevalent in inflammatory lesions and play a critical role in directing the inflammatory response.

In the conventional understanding, macrophages are recognized as immune cells with phagocytic abilities and evolutionary conservation. However, recent research has unveiled tissue‐resident macrophages originating from embryonic precursors, present in various organs even before birth. These tissue‐resident macrophages, beyond their phagocytic functions, exhibit specialized roles in specific organs. For example, microglia in the brain facilitate synaptic remodelling during development, while adipose tissue macrophages regulate thermogenesis.^5^ In the spleen, red pulp macrophages are responsible for recycling damaged red blood cells.^6^ The heart, being a metabolically demanding organ, undergoes comprehensive inflammatory responses to stress and injury, involving various immune cells. Macrophages play a pivotal role in this cascade, producing pro‐inflammatory cytokines that contribute to tissue damage and myocardial remodelling under hypoxic conditions.^7^

Have recent studies elucidated specialized functions of CRMs beyond immunity? By employing advanced techniques such as single‐cell sequencing and genetic lineage tracing, researchers have unveiled a distinct subset of cardiac innate immune cells with unique origins, proliferation mechanisms and roles distinct from monocyte‐derived macrophages. Some of these macrophages colonize the heart during embryonic development, deriving from precursor cells in the yolk sac and fetal liver, and maintain their population through localized proliferation, known as CRMs. This review aims to introduce their origin and characteristics, delve into their role in cardiac homeostasis and explore their implications in the pathogenesis of cardiovascular diseases, potentially paving the way for innovative therapeutic strategies.

## 2 ORIGIN AND PHENOTYPIC CLASSIFICATION OF CRMs

**FIGURE 1:**
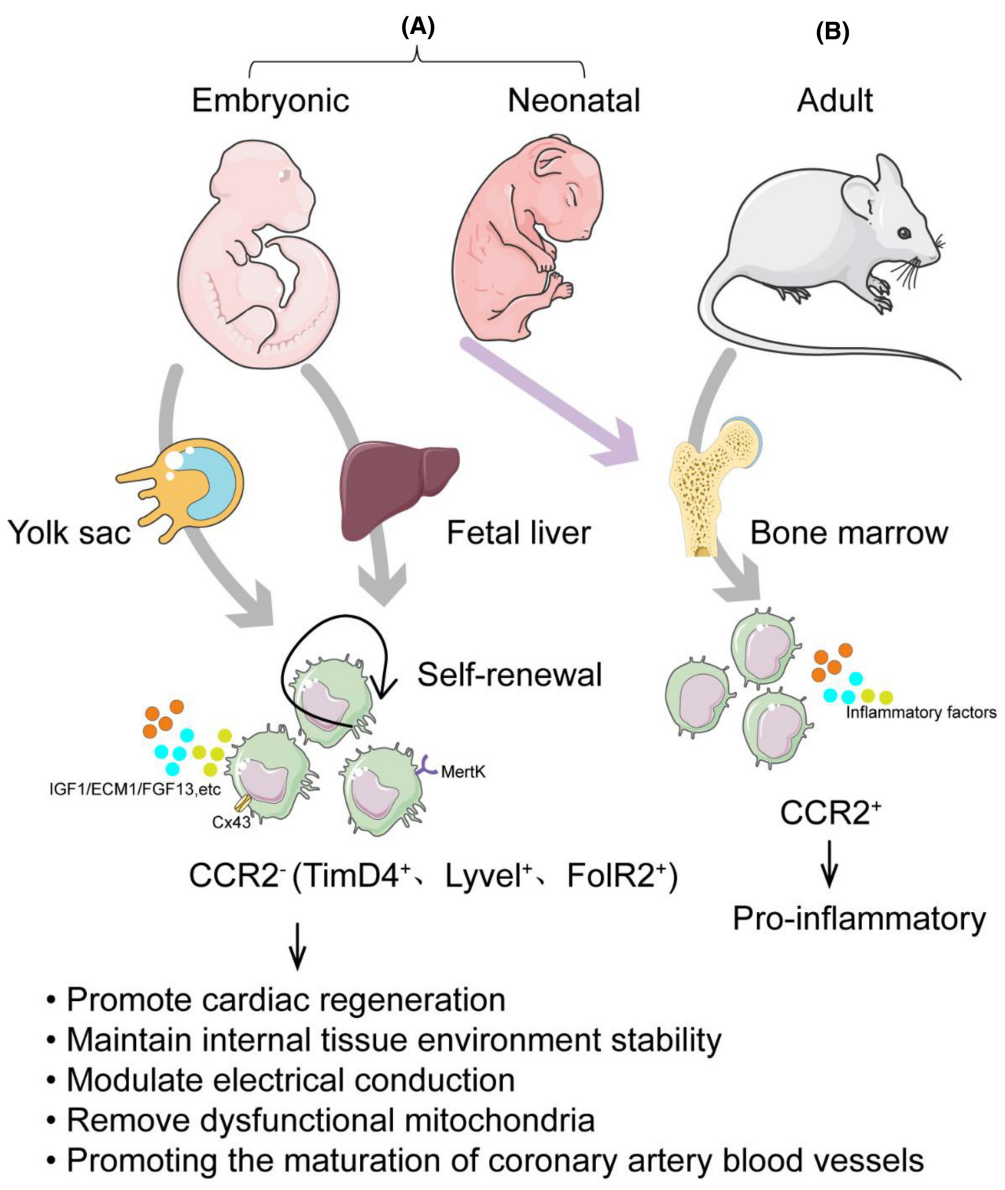
Categorization and origins of CRMs. The categorization and origins of CRMs are delineated based on their expression of C‐C chemokine receptor 2 (CCR2). Under physiological conditions within the heart, two primary macrophage subtypes emerge: Those derived from embryonic origins and those originating from the bone marrow. Embryonic‐derived macrophages: These macrophages originate from the yolk sac and fetal liver during embryonic development. They express CCR2‐ and possess the capacity for self‐renewal, thus designated as CRMs. Research indicates that these macrophages predominantly manifest MHCII^lo^ and CCR2^−^ expression and exhibit characteristic marker gene profiles, including TimD4^+^, Lyve1^+^ and FolR2^+^, hence denoted as TLF^+^ macrophages (A). Bone marrow‐derived macrophages: These macrophages stem from mature monocytes and express CCR2^+^. In the early stages of development, CCR2^+^ macrophages migrate into the heart and sustain their population through continual recruitment and proliferation of monocytes. In contrast to embryonic‐derived macrophages, these CCR2^+^ macrophages demonstrate a propensity toward pro‐inflammatory functions (B).

In a quiescent state, the heart harbours diverse macrophage populations originating from various sources, distinguishable by the expression of C‐C chemokine receptor 2 (CCR2) on their cell surface, delineating their embryonic or bone marrow lineage (Figure 1). Embryonic macrophages, emerging from the yolk sac and fetal liver during organogenesis, dynamically exhibit CCR2 expression. In the adult mouse heart, resident macrophages comprise around 6% to 8% of non‐myocytes, enveloping each cardiomyocyte with an average of five resident macrophages.^8^ The CCR2^−^‐ macrophage population within the cardiac milieu demonstrates a remarkable capacity for self‐renewal, thereby earning them the distinguished title of CRMs. These specialized macrophages originate from two distinct sources, namely the yolk sac and the fetal liver. As the embryonic development of mice progresses, precursor cells present in the yolk sac are selectively recruited to the developing heart around E11.5. Once in the heart, these cells undergo a process of differentiation, giving rise to CCR2^−^MHC‐II (major histocompatibility complex II) macrophages characterized by low expression levels. Intriguingly, these CCR2^−^MHC‐II^low^ macrophages are strategically implanted beneath the epicardium and actively participate in the intricate process of coronary artery development and maturation during the critical time window spanning from E13.5 to E14.5,^9^ the study provided additional details on the expression of three marker genes, namely TimD4^+^, Lyvel^+^ and FolR2^+^, in CCR2^−^MHC‐II^low^ CRM. Consequently, the term TLF^+^ macrophages was employed to describe this specific cellular group.^10^ During the initial 7‐day period following birth in mice, the dominant source of CRM can be traced back to the yolk sac, which actively contributes to the restoration or renewal of the cardiac tissue. Conversely, macrophages derived from mature monocytes demonstrate the expression of CCR2^+^. At early stages of development, CCR2^+^ macrophages infiltrate the cardiac region and maintain their cellular count via persistent recruitment and proliferation of monocytes, thereby exhibiting heightened pro‐inflammatory capacities.^11^

CCR2 serves as an authentic biomarker indicating the origin of blood monocytes and exhibits significant enrichment within NLRP3 inflammasomes. Functionally, this chemokine receptor assumes a pivotal role as a chemotactic factor receptor, critically regulating the process of cellular migration.^12^ In an effort to further refine macrophage subtypes, researchers have proposed the incorporation of additional surface markers for the purpose of classification. In a recent investigation,^10^ by employing a combination of cell tracking and single‐cell RNA sequencing (scRNA‐seq), a profound delineation of four transcriptionally distinct macrophage populations exhibiting discrete temporal cycles and specialized functionalities within the healthy adult myocardium was observed. These four populations encompass a unique subgroup (TIMD4^+^LYVE1^+^MHC‐IICCR2^−^) that operates independently of blood monocytes while retaining the capacity for self‐regeneration (referred to as the TIMD4 cluster). Furthermore, a subgroup (TIMD4^−^LYVE1^−^MHC‐II^hi^CCR2^−^) was identified, which exhibited a partial displacement by monocytes (referred to as the MHC‐II cluster), and two additional subgroups (TIMD4^−^LYVE1^−^MHC‐II^hi^CCR2^+^) that were entirely replaced by monocytes (referred to as the CCR2 cluster and the ISG cluster, respectively). CRMs possess the ability to express a diverse array of pivotal reparative genes, a characteristic that is absent in recruited macrophages. These genes, including Timd4, Lyve1, Igf1, among others, play a significant role in facilitating the reparative functions of these cells. Of notable importance is the phosphatidylserine receptor known as TIMD4, which actively participates in the cell clearance process.^13^ Deficiency in the expression of TIMD4 further amplifies the detrimental effects of ischemic injury;^14^ LYVE1 exerts a crucial role in maintaining vascular homeostasis by binding to hyaluronic acid expressed on the surface of smooth muscle.^15^ In contrast, IGF1 directly stimulates angiogenesis^16^ and regulates remodelling of the coronary artery.^17^ Regarding TIMD4LYVE‐1, these molecules predominantly localize near endothelial cells,^18^ facilitating the transcription of angiogenesis‐related genes and inducing the formation of endothelial cell tubes. Moreover, they directly interact with smooth muscle cells,^15^ thus ensuring the proper tension of arterial smooth muscle. Irrespective of their origin as resident or recruited macrophages, both subsets exhibit significant heterogeneity, complexity and adaptability during steady‐state and pathological conditions. Similarly, human cardiac macrophages can be categorized into three distinct groups according to their expression levels of HLA‐DR (human homologue of MHC‐II) and CCR2: CCR2^+^HLA‐DR^low^, CCR2^+^HLA‐DR^high^ and CCR2‐HLA‐DR^high^ cells.^19^ MertK, a conserved marker for macrophages in both humans and mice, is used for the detection of CCR2^+^HLA‐DR^high^ and CCR2^−^HLA‐DR^high^ cells.^20^, ^21^ Conversely, CCR2^+^HLA‐DR^low^ cells show negative staining, indicating their monocytic characteristics. Regarding morphology, CCR2^+^HLA‐DR^high^ and CCR2^−^HLA‐DR^high^ macrophages demonstrate increased granularity, with CCR2^+^HLA‐DR^high^ cells exhibiting a larger size in comparison to CCR2^−^HLA‐DR^high^ cells. These macrophages express numerous markers, including MERTK, SIGLEC1, MRC1, LYVE1, MAF, TREM2, CD16, CD32, SPP1/Osteopontin and MARCO. The genes upregulated in these macrophages are notably enriched in K‐RAS, IL6/STAT3, IL2/STAT5 and inflammatory pathways. Conversely, the upregulated genes specifically in CCR2^+^HLA‐DR^high^ macrophages display significant enrichment in signalling pathways associated with inflammation, such as TNF/NFκB signalling, inflammatory response, allograft rejection, IL2/STAT5, IL6/STAT3, interferonγ, hypoxia and K‐RAS signalling. Furthermore, these macrophages manifest chemokines, chemokine receptors and mediators of IL1, NFκB and IL6 signalling. Notably, in the presence of apoptotic cardiomyocytes, activation of CCR2^+^HLA‐DR^high^ macrophages via the TLR9 pathway, which relies on the release of mitochondrial DNA, promotes the release of neutrophil chemoattractants.^22^ However, the precise functions of CCR2^+^HLA‐DR^high^ macrophages in the steady‐state heart remain incompletely understood. In contrast, CCR2^−^HLA‐DR^high^ macrophages are found to be abundant in epithelial mesenchymal transition, coagulation, myogenesis, p53 and IL2/STAT5 signalling. Furthermore, they exhibit higher expression levels of growth factors, extracellular matrix complexes and signalling genes associated with tissue repair. CCR2^−^HLA‐DR^high^ macrophages contribute to the enlargement of the left ventricle and the expansion of the coronary artery system at both the macro‐ and microvascular levels, effectively maintaining an adequate cardiac output despite reduced contractility. These macrophages establish stable interactions with adjacent cardiomyocytes through adhesive plaque complexes and can sense mechanical stretching, which in turn promotes the expression of pro‐angiogenic growth factors.^23^ This suggests that CCR2^+^ promotes an inflammatory response, while CCR2^−^ contributes to the maintenance of tissue homeostasis. CCR2^−^ is capable of establishing gap junction connections with cardiomyocytes, thereby regulating their resting and action potentials,^24^ as well as participating in atrioventricular conduction.^25^ A recent study has presented evidence that resident macrophages residing in the heart actively eliminate dysfunctional mitochondria released by cardiomyocytes, thus preserving cardiac health.^26^ Despite this finding, the exact mechanism by which immune cells manipulate mitochondrial quality control in infectious cardiac conditions remains largely unknown. However, a recent investigation offers some insights. By identifying a distinct subset of resident macrophages titled CD163^+^RETNLA^+^(Mac1),^27^ this study found that these cells undergo self‐renewal during sepsis. Notably, this specific population of cells exhibits robust phagocytic activity aimed at eliminating dysfunctional mitochondria associated with impaired cardiac function. The primary cell surface markers of cardiac macrophages are summarized in Table 1.

**TABLE 1:** Comparative surface markers of human and mouse macrophages.

Species	Universal markers	Markers	Ref (PMID)
Mouse	CD45^+^F4/80^+^CD11b^+^	CCR2^−^Ly6c^−^MHC‐II^Hi^CX3CR1^hi^CD206^int^CD11c^lo^	24439267
		CCR2^−^Ly6c^−^MHC‐II^lo^CX3CR1^int^CD206^hi^CD11c^lo^	
		CCR2^−^Ly6c^+^	
		CCR2^+^Ly6c^hi^	
	CD45^+^CD64^+^CD11b^+^	TIMD4^+^LYVE1^+^MHC‐II^lo^CCR2^−^(TIMD4 cluster)	30538339
		TIMD4^−^LYVE1^−^MHC‐II^hi^CCR2^−^(MHC‐II cluster)	
		TIMD4^−^LYVE1^−^MHC‐II^hi^CCR2^+^(CCR2 cluster)	
		TIMD4^−^LYVE1^−^MHC‐II^hi^CCR2^+^(ISG cluster)	
Human	CD45^+^CD64^+^CD14^+^	CCR2^−^MHC‐II^hi^	30538339
		CCR2^+^MHC‐II^hi^	
		CCR2^+^MHC‐II^lo^	
	CD45^+^CD14^+^CD64^+^CD68^+^	CCR2^+^HLA‐DR^lo^	29892064
		CCR2^+^HLA‐DR^hi^	
		CCR2^−^HLA‐DR^hi^	
	CD45^+^CD11b^+^	F4/80^+^CD163^+^RETNLA^+^TREM2^hi^	36635449
		F4/80^−^LY6C^+^	
		F4/80^+^CD163^−^RETNLA^−^	

## 3 EPIGENETIC REGULATION OF CARDIAC MACROPHAGE PHENOTYPIC TRANSFORMATION IN CARDIAL REPAIR

Over the past decade, numerous epigenetic factors, including DNA methylation, histone post‐translational modifications and non‐coding RNAs, have emerged as crucial regulatory mechanisms of immune cell phenotypes. Increasing evidence underscores the significance of epigenetics in dynamically regulating critical signalling pathways that can alter monocyte/macrophage phenotypes in response to environmental stimuli, thereby influencing the pathophysiology of cardiovascular diseases.

Studies have assessed the DNA methylation status of gene‐specific promoters related to M1/M2 polarization markers in peripheral blood mononuclear cells (PBMCs) of patients with coronary artery disease (CAD). Compared to control groups, CAD patients exhibit distinct patterns of gene‐specific promoter DNA methylation. Significant differences in methylation percentages were observed in genes such as *STAT1*, *IL12b*, *MHC2*, *iNOS*, *JAK1* and *JAK2* between CAD patients and controls. DNA methylation‐related pharmacological interventions may provide new therapeutic possibilities for atherosclerotic diseases.^28^ Similarly, Paahuleva et al. demonstrated that miRNA levels in PBMCs differ between myocardial infarction (MI) patients and healthy controls. In MI patients, miR‐143 and miR‐145 levels were upregulated in PBMCs, implicating their role in macrophage differentiation and activation.^29^ Additionally, miR‐155 is elevated in macrophages of the injured heart, and its upregulation directly impacts fibroblast proliferation during post‐infarction remodelling. When miR‐155 is downregulated in macrophages, the expression of CCL2, a chemokine recruiting monocytes, decreases, suggesting that inhibiting macrophage‐derived miR‐155 expression may attenuate the fibrotic inflammatory response in damaged myocardium.^30^ Recent studies have validated the upregulation of NPM1 mRNA and protein levels in PBMCs of MI patients. IL‐4‐induced oligomerized NPM1 recruits KDM5b to form an epigenetic complex at the TSC1 promoter, mediating the erasure of H3K4me3 modification and thus inhibiting TSC1 gene transcription.^31^ NPM1‐deficient cardiac macrophages exhibit an enhanced reparative phenotype and function without pro‐inflammatory characteristics. Covarrubias et al. identified macrophage‐derived lncRNAs such as Cox2 and AK170409 using CRISPR/Cas‐based screening techniques. These genes act as upstream regulators of NF‐κB, participating in its signalling pathway. Knockout of these lncRNAs significantly reduces the expression of pro‐inflammatory genes.^32^ Recent findings revealed that the non‐coding RNA yREX3 from human extracellular vesicles mitigates cardiac ischemic injury through selective DNA methylation. yREX3 interacts with PTBP3 to methylate the Pick1 gene locus in a DNA methyltransferase‐dependent manner, resulting in its epigenetic silencing. Inhibition of Pick1 in macrophages enhances Smad3 signalling, thereby reducing myocardial necrosis in rats with myocardial infarction.^33^ Smad3 can enhance efferocytosis^34^ and has been shown to enable high‐throughput cardiogenesis^35^ and is a major effector of TGF‐β's influence on macrophage function in vivo and in vitro. However, this study demonstrates that yREX3‐mediated Smad3 activation is independent of TGF‐β secretion.

In addition to ncRNAs, inhibition of histone deacetylase (HDAC) activity also protects left ventricular function and myocardial remodelling post‐myocardial infarction. HDAC inhibition can regulate macrophage phenotype and promote early resolution of inflammation to maintain left ventricular function post‐MI. During the early inflammatory healing phase (30 h), there is an increase in CD45^+^/CD11b^+^/CD206^+^ M2 reparative macrophages, providing a foundation for improved ventricular function and reduced adverse remodelling of the infarcted myocardium in later stages.^36^

These research findings collectively demonstrate that epigenetics, through the regulation of inflammatory responses and immune cell phenotypes, particularly macrophages, influence myocardial healing and ventricular function post‐MI. This provides new avenues for repairing MI damage and improving patient outcomes. This review summarizes the epigenetic regulation of macrophage polarization in cardiovascular diseases in detail.^37^ The intensity of the inflammatory response and macrophage polarization depends on the cells' ability to respond to environmental changes and stimuli. Epigenetic mechanisms provide pathways for such adaptive responses and enhance macrophage diversity and polarization vigour. Therefore, in future research, epigenetics will serve as an effective pathway to regulate immune cells and pathological processes such as inflammatory responses, offering new solutions for cardiac metabolism and cardiovascular diseases.

## 4 CARDIAC DEVELOPMENT AND HOMEOSTASIS

Indeed, the recruitment of embryonic macrophages synchronizes with significant morphological changes observed in cardiac development, including the formation of the ventricular septum, the development and remodelling of endocardial cushions and valves, the growth and maturation of myocardium, the establishment of the conduction system and the emergence and expansion of coronary arteries and lymphatic vessels. Therefore, the macrophages residing in the cardiac tissue are deemed crucial contributors to these processes. Their functional roles encompass the phagocytosis of dying cells, the secretion of soluble cytokines, the interaction with progenitor cells, the recruitment of progenitor cells and the potential to differentiate into alternative cell types.

**FIGURE 2:**
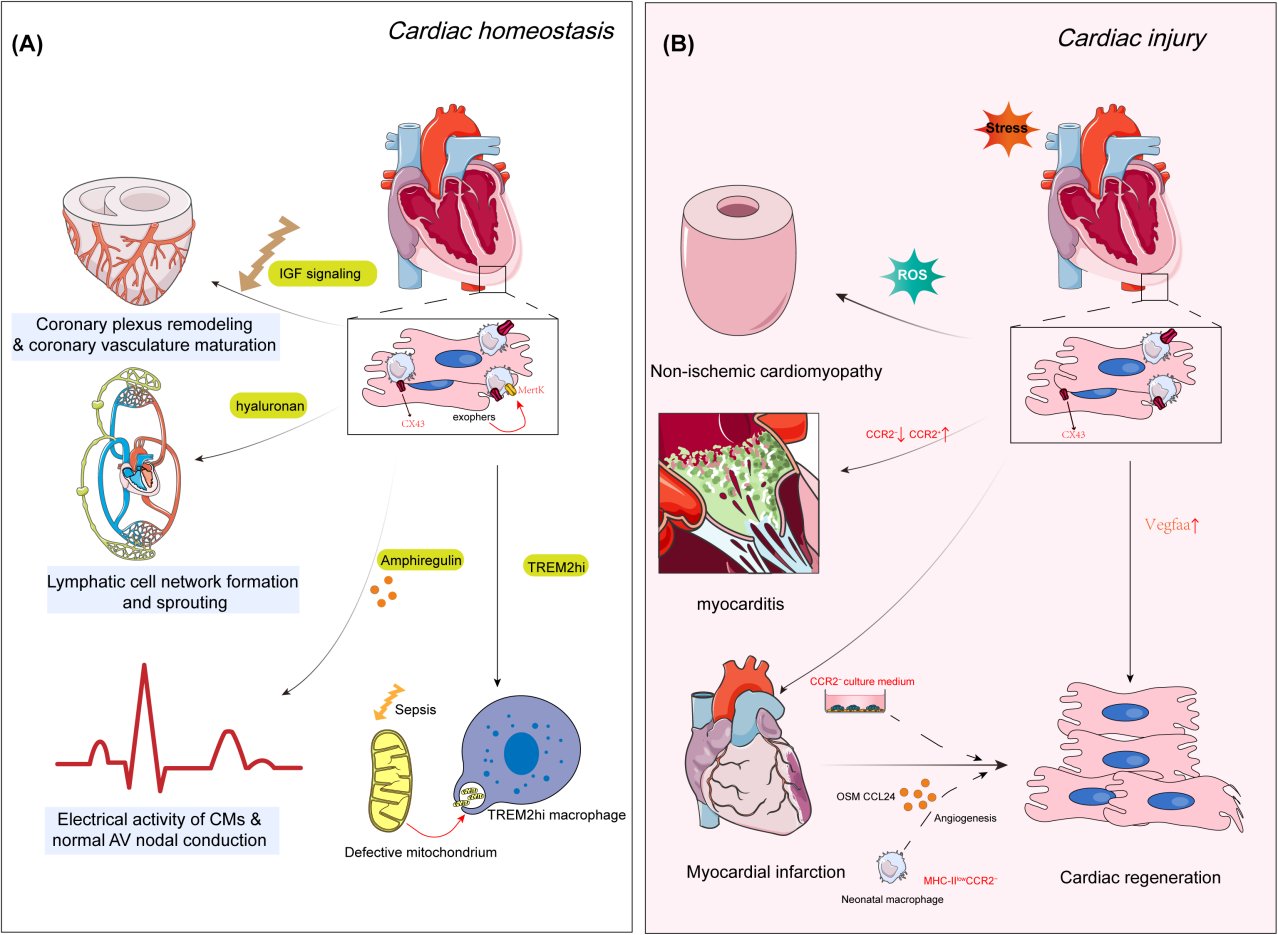
Role of cardiac macrophages in cardiac development, homeostasis and response to injury. Under stable conditions, these macrophages help maintain the heart's internal environment. CCR2^−^ macrophages promote vascular maturation in coronary arteriogenesis through vasodilation, potentially mediated by IGF1 and IGF2. They also support lymphatic capillary development in the heart and prevent cardiac conduction issues. Resident macrophages play a key role in maintaining mitochondrial homeostasis, clearing dysfunctional mitochondria to enhance cardiac function (A). They also contribute to vascular oxidative stress and may vary in their impact on myocarditis. Macrophages participate in cardiac regeneration processes through vegfaa expression. Neonatal macrophages facilitate myocardial regeneration through myocardial cell proliferation and angiogenesis (B).

In a state of homeostasis (Figure 2), resident macrophages play a significant role in preserving the stability of the cardiac microenvironment. As previously mentioned, macrophages expressing CCR2^−^, derived from the yolk sac, actively facilitate the functional maturation of coronary arteries through a process known as arteriogenesis. This process involves the dilation of arterial vessels, which ultimately promotes the development of a mature and fully functional coronary artery network. Furthermore, compelling evidence suggests that Insulin‐like Growth Factors 1 and 2 (IGF1 and IGF2) may serve as potential mediators in the complex process of coronary artery vascularization. Notably, the absence of CCR2‐expressing macrophages during embryonic development leads to perturbations in the normal progression of cardiac development and compromises vascular maturation.^16^ However, the function of this subset of macrophages in the adult heart is not yet clear. Other evidence suggests that macrophages have close interactions with newly formed lymphatic capillaries in the developing heart. Firstly, macrophages are found to distribute and migrate in the epicardial subepicardial cavity of the developing heart, corresponding to the appearance of new lymphatic vessels.^38^ Furthermore, the absence of macrophages results in extensive vascular disruption, leading to shortened and irregularly patterned lymphatic vessels in the heart. Mechanistically, macrophages secrete hyaluronic acid, which is necessary for lymphatic sprouting through direct interactions with lymphatic endothelial cells. Apart from their interaction with blood vessels, CRMs also communicate with cardiomyocytes through cellular projections. Cardiomyocytes regulate cardiac contraction precisely through excitation‐contraction coupling and coordinate this process with neighbouring cells via gap junctions. Growing evidence supports the notion that macrophages can prevent cardiac conduction block and ventricular arrhythmias through both direct and indirect mechanisms. Studies using Cx3cr1GFP/+ transgenic mice have demonstrated that macrophages are abundantly present in the atrioventricular node in both human and mouse hearts, with a higher cell density in the left ventricle. These macrophages exhibit a high expression of genes related to cardiac conduction, including Cx43, and establish electrical coupling with cardiomyocytes in the distal atrioventricular node through gap junctions that contain Cx43. Specific knockout of Cx43 in CX3CR1^+^ cells leads to atrioventricular conduction abnormalities, but not atrioventricular block as observed in Cd11bDTR‐mediated macrophage depletion,^25^ this phenomenon could be attributed, at least in part, to the modulation of cardiac myocyte gap junction proteins, specifically Cx43, which are regulated by amphiregulin derived from macrophages. This intricate control mechanism serves to uphold the integrity of electrical impulse conduction within the heart.^39^ However, the role of macrophages in conduction abnormalities beyond the atrioventricular node, including conditions such as atrial fibrillation, ventricular arrhythmias, genetic conduction disorders and conduction system development, remains unclear. Recent investigations unveil a notable expansion of inflammatory monocytes and SPP1^+^ macrophages in patients with atrial fibrillation. The stromal cell protein SPP1 is found to be upregulated in the bloodstream of individuals with atrial fibrillation, thereby stabilizing collagen and fostering fibrosis in hypertensive patients. The authors posit that the heightened presence of SPP1 originating from macrophages contributes to the heterogeneity of atrial tissue, thus impeding intercellular communication among cardiomyocytes.^40^ A significant research discovery is the identification of extracellular vesicles called exophers, which are released by healthy myocardial cells and play a critical role in maintaining mitochondrial homeostasis. These exophers are readily absorbed and processed by phagocytic cells, particularly those expressing the phagocytic receptor Mertk, located around cardiac myocytes. This process ensures the health of cardiac mitochondria and myocardial cells by eliminating cellular waste, including dysfunctional mitochondria. Interestingly, defects in Mertk lead to mitochondrial accumulation despite an elevated number of macrophages present. Using a combination of single‐cell RNA sequencing and fate mapping in a sepsis mouse model, the authors have demonstrated that the Mac1 subset exhibits a distinct transcriptional profile enriched for phagocytic activity and shows high expression of TREM2 (TREM2^hi^). TREM2^hi^ Mac1 cells actively clear dysfunctional mitochondria released by myocardial cells,^27^ they also suppress mitochondrial SLC25A53 expression, resulting in increased production of itaconic acid, which can inhibit myocardial cell apoptosis and improve cardiac function.^41^ RCM also function as metabolic stress‐response cells. In ApoE knockout mice fed a high‐fat diet, RCM display upregulated endoplasmic reticulum stress, unfolded protein response and apoptosis‐related pathways compared to infiltrating macrophages.^42^

## 5 CRMs AND CARDIOVASCULAR DISEASES

A comprehensive overview of experimental studies focused on macrophages in cardiovascular disease can be found in Table 2.

**TABLE 2:** Experimental studies on macrophages in cardiovascular diseases: An overview.

	Model	End point	Target	Findings	Refs(PMID)
Myocardial injury	Myocardial infarction, diphtheria toxin (DT) cardiomyocyte ablation and syngeneic heart transplantation	Distinguish tissue‐resident from recruited cell populations	CCR2^−^ and CCR2^+^ macrophages, MYD88 signalling, intravital imaging, cardiac function	Monocytes and macrophages derived from them invade the heart following cardiomyocyte damage and mostly take over the populations of resident tissue macrophages	30582448
	Myocardial ischemia reperfusion	Role of phagocytic clearance by resident and recruited phagocytes after myocardial ischemia reperfusion	Infarct size and cardiac function	Mertk deficiency in macrophages resulted in reduced cardiac wound cleaning, increased infarct size and impaired cardiac function	28851810
	Permanent coronary artery ligation	Evaluation of transcriptome of macrophages were isolated from the infarct region at days 1, 3 and 7 post‐MI	Transcriptome analysis, in situ hybridization	Macrophages exhibit unique gene expression patterns during the initial week of myocardial infarction, and metabolic reprogramming plays a crucial role in their polarization	29868933
	Multiple transgenic mice, cardiac tissues of patients with ischemic cardiomyopathy and healthy control subjects	Role of Lgmn derived from cardiac resident macrophages in MI	Infarct size, cardiac function, anti‐inflammatory mediators and proinflammatory mediators	Lgmn deficiency led to a significant worsening of cardiac function	35430895
	Permanent coronary occlusion and clinically relevant ischemia and reperfusion, cultured primary macrophages	Role of myeloid Vegfc	Transcriptome analysis, infarct size and cardiac function	Cardiac macrophages facilitate healing by encouraging myocardial lymphangiogenesis and reducing inflammatory cytokines	35271504
	Neonatal mice (P14) and adult mice (>12 weeks)	Discriminating functions within mixed populations	Single‐cell transcriptomics, infarct size and cardiac function	TIMD4 could serve as a reliable lineage marker for a specific subset of resident cardiac macrophages	30538339
	Adult mice (>12 weeks)	Evaluation of diverse macrophages populations	In vivo cell tracking, parabiosis, bone marrow transplants and fate mapping	The adult mammalian heart harbours distinct subsets of macrophages originating from both embryonic and adult sources, with the former sustained through local proliferation and the latter through replacement by blood monocytes	24439267
	Primary mouse cardiomyocytes, neonatal mice (<7 days)	Role of Tβ4‐loaded cardiac‐resident macrophage‐derived extracellular vesicles modified with monocyte membranes	Infarct size and cardiac function	Tβ4‐MmEVs stimulated cardiomyocyte proliferation and enhanced endothelial cell migration	37597679
	Adult mice (>12 weeks)	Evaluation of cardioprotective effects of hydrogen sulfide (H2S)	Infarct size and cardiac function	H2S might serve as a potential therapeutic agent for myocardial infarction by promoting M2 macrophage polarization	27296720
Non‐ischemic cardiomyopathy	Male wild‐type mice (C57 BL/6 J) and those overexpressing human superoxide dismutase (6‐TgN(SOD1)3Cje) (SOD‐TG), hypertensive patients	Role of CCR2 expression on monocytes in hypertension‐induced vascular remodelling	MCP‐1, CCR2 and BNP, plasma measurements	CCR2 expression in monocytes is crucial for vascular inflammation and remodelling in Ang II‐induced hypertension	15059935
Non‐ischemic cardiomyopathy	Sham or TAC‐operated mice	Determine the identity and abundance of immune cells in the heart at 1 and 4 weeks after TAC	Cytometry by time‐of‐flight, single‐cell transcriptomics	Cardiac resident macrophages are a diverse group of immune cells that play essential roles in promoting angiogenesis and preventing fibrosis in response to cardiac pressure overload	34645281
	C57BL/6 male mice	Role of CCR2^+^ monocyte‐derived cardiac macrophages for adverse LV remodelling	Proinflammatory cytokine, LV systolic function	CCR2^+^ monocytes/macrophages may represent key targets for immunomodulation to delay or prevent HF in pressure‐overload states	30062209
	C57BL/6 male mice	Role of myelomonocytic cells in mediating arterial hypertension	Bloodpressure, vascular endothelial and smooth muscle function, NADPH oxidase subunits gp91phox and p67phox	CD11b^+^Gr‐1^+^ monocytes require the presence of gp91phox and Agtr1 to facilitate ATII‐induced arterial hypertension, vascular dysfunction and inflammation, oxidative stress and the suppression of NO/soluble guanylyl cyclase/cGMP activity	21875910
	Male C57BL/6 mice	Role of cardiac macrophages during cardiac fibrosis	Single‐cell transcriptomics, lineage tracing and parabiosis	ALKBH5‐mediated m6A modification of IL‐11 promotes the transition of macrophages to myofibroblasts and leads to pathological cardiac fibrosis in mice	33128959
Myocarditis	Murine ICI myocarditis model (Ctla4^+/‐^Pdcd1^−/−^mice)	Role of macrophages in Immune checkpoint inhibitors myocarditis	Single‐cell RNA‐sequencing, immunostaining, flow cytometry, in situ RNA hybridization, molecular imaging and antibody neutralization	ICI myocarditis is linked to the expansion of a distinct group of IFN‐γ‐induced inflammatory macrophages	37746718
	Murine experimental autoimmune myocarditis (EAM)	Role for cardiac fibroblasts in facilitating monocyte‐to‐macrophage differentiation of both Ly6C^hi^ and Ly6C^lo^ cells	Parabiosis, microarray gene expression	IL‐17A trans‐signalling encourages monocyte‐derived macrophages to adopt a proinflammatory phenotype	31269438
	Nox4^flox/flox^ mice	Role of NOX4 regulates resident macrophage‐mediated inflammation and diastolic dysfunction in stress cardiomyopathy	IL18, IL6, CCL2 and TNFα	Excessive sympathetic stimulation activates resident macrophages (CCR2^−^MHCII^+^), leading to myocardial inflammation, fibrosis and impaired diastolic function through CM NOX4‐dependent ROS	37871532
	Male wild‐type mice, mouse peritoneal macrophages	Role of SA1 in obesity may cause hypertension in macrophages.	Blood pressure, vascular endothelial growth factor B	Abnormal PVAT in obesity might contribute to hypertension through VEGF‐B‐induced vascular dysfunction. SR‐A1 appears to counteract this by suppressing VEGF‐B production in macrophages	
	Lewis rats	Evaluation of preventive effects of cyclosporine, prednisolone and aspirin on autoimmune giant cell myocarditis in rats	Heart weight/body weight, lung weight/body weight and liver weight/body weight ratios	Cyclosporine administration can avert autoimmune myocarditis, whereas standard doses of prednisolone or aspirin are ineffective in preventing this condition	8459085
	Rat giant cell myocarditis (GCM), human heart sample	Role of NETosis in GCM pathogenesis	Single‐cell RNA transcriptome, MPO, H3cit, mass cytometry	Suppression of NETosis through PAD4 inhibition reduced inflammatory responses in giant cell myocarditis	37534129
Cardiac regeneration	Larval zebrafish	Role of macrophages in zebrafish cardiac regeneration	Heartbeat‐synchronized live imaging, RNA sequencing and macrophage‐null genotypes	The activation of the epicardium, facilitated by macrophages, is essential for promoting cardiomyocyte multiplication	35688158
	AR‐injured neonatal mouse	Role of murine neonatal cardiac macrophage in adult cardiac repair	pH 3, cardiac function	Neonatal cardiac macrophages possess the capacity to promote regeneration	32055006
	Neonatal MI model and clodronate liposome treatment	Evaluation of macrophages for neonatal heart regeneration	Scar formation, pH 3	Angiogenesis and cardiac regeneration in neonatal mice are facilitated by crucial signals emitted from macrophages	24569380
	In vivo cardiomyocyte cell ablation model	Investigation of distinct macrophage lineages contribute to disparate patterns of cardiac recovery and remodelling in the neonatal and adult heart	Flow cytometry, genetic lineage tracing, inflammatory response, TNFα and IL1β, cardiac function	In normal conditions, the mature heart harbours macrophages of embryonic origin with comparable characteristics. However, following cardiac injury, these cells are supplanted by inflammatory monocytes and their macrophage derivatives. These newcomers have restricted ability to foster cardiac healing and instead contribute to inflammation and oxidative damage	25349429
	In vitro engineered cardiac tissue	Investigation of crosstalk between macrophage and human pluripotent stem cell‐derived cardiomyocytes	3D inverted invasion assay	The secretome of cardiomyocytes (CMs) has the capacity to attract pro‐inflammatory (M1) macrophages, a process partially mediated by BMP4 released from CMs	26103914
	In vitro engineered cardiac tissue	Role of macrophage in cardiac reconstruction	Single‐cell RNA transcriptome	In response to the 3D scaffold microenvironment, cells adaptively reverted to a less specialized state to ensure survival, resulting in the formation of diverse tissue types	31455969
	C57BL/6 male mice	Role of CCR2^−^ macrophages in the chronically failing heart	Cardiac function, IGF1, Nppa, Nppb and Myh7, mitochondrial function, TRPV4 channel	Macrophages expressing CCR2 formed connections with adjacent heart muscle cells through focal adhesion structures. These macrophages became activated when subjected to mechanical stretching, a process mediated by a pathway dependent on transient receptor potential vanilloid 4 (TRPV4), which regulated the expression of growth factors	34320366
	C57BL/6 male mice	Discriminating distinct molecular signature of F4/80^hi^ and CD11b^hi^ MΦ	Transcriptional analysis, serum protein	Proper maturation of macrophages (MΦ) is heavily dependent on the transcription factor Irf8	27412700
	Macrophages generated from human induced pluripotent stem cells (iMϕs)	Evaluation of characteristics of macrophages generated from human induced pluripotent stem cells (iMϕs)	Morphology, pro‐inflammatory (IL‐6, CXCL8, CCL2, CCL4, CXCL1, CXCL10) and anti‐inflammatory (IL‐10, IL‐1RA, CCL22) cytokines	iMϕs represent a class of differentiated macrophages that exhibit a “naïve‐like” state, characterized by low activation and minimal polarization. These cells retain the capacity to mount robust inflammatory and antibacterial responses when challenged with inflammatory stimuli or pathogenic agents	32582159
	iPSC‐derived macrophage‐like cells	Evaluation of characteristics of macrophages generated from human induced pluripotent stem cells (iMϕs)	Macrophage function	Macrophages generated from induced pluripotent stem cells (iPSCs) demonstrate a robust cellular profile closely resembling that of primary macrophages. These iPSC‐derived cells successfully replicate essential functional attributes, including the secretion of cytokines, the ability to engulf particles through phagocytosis and the capacity for directed movement in response to chemical stimuli (chemotaxis)	32645954
Cardiac aging	Young (3‐month‐old), middle‐aged (15‐month‐old) and old (23‐month‐old) CB6F1 mice	Evaluation of the effects of aging on left ventricular (LV) geometry, collagen levels, matrix metalloproteinase (MMP) and tissue inhibitor of metalloproteinase (TIMP) abundance and myocardial fibroblast function.	Myocardial matrix metalloproteinase profiles and fibroblast function	The impact of senescence on left ventricular dimensions, extracellular matrix composition, proteolytic enzyme balance and fibroblast activity	15820210
	Young (6–9 months), middle‐aged (12–15 months), old (18–24 months) and senescent (26–34 months) mice	Evaluation of cardiac aging signature	Bcl6, Ccl24 and Il4; MMP‐9	Matrix metalloproteinase‐9 (MMP‐9) contributes to the age‐related inflammatory profile through both direct and indirect mechanisms that influence macrophage polarization states	25883218
	Young (6–9 months), middle‐aged (12–15 months), old (18–24 months) and senescent (26–34 months)	Role of MMP‐9 in cardiac aging	LV function, CTGF, TGF‐β	As the aging process progressed, both connective tissue growth factor (CTGF) and transforming growth factor‐beta (TGF‐β) exhibited elevated levels	22918978
	Adult (7.5 ± 0.5 months old) and senescent (31.7 ± 0.5 months old) C57/BL6J mice	Investigation of plasma biomarkers of cardiac aging	Macrophage functions, MMP‐9 and MCP‐1	Monocyte chemoattractant protein‐1 (MCP‐1) and matrix metalloproteinase‐9 (MMP‐9) may serve as circulating biomarkers indicative of cardiac senescence	21685172
	Adherent splenocytes	Evaluation of the ability of macrophage to change phenotypes in response to environmental factor	(iNOS), IL‐6, IL‐1β and TNF‐α	Dysfunctional macrophage polarization in older adults may disrupt the regulation of the host immune response	22175541
	Peripheral blood monocytes from 159 subjects in 2 age categories, 21–30 and >65 years of age	Effects of aging on human TLR function	TNF‐α, IL‐6, TLR2/6	Elderly individuals exhibit diminished cytokine responses to TLR1/2 stimulation	17202359

### 5.1 CRMs and myocardial infarction

**FIGURE 3:**
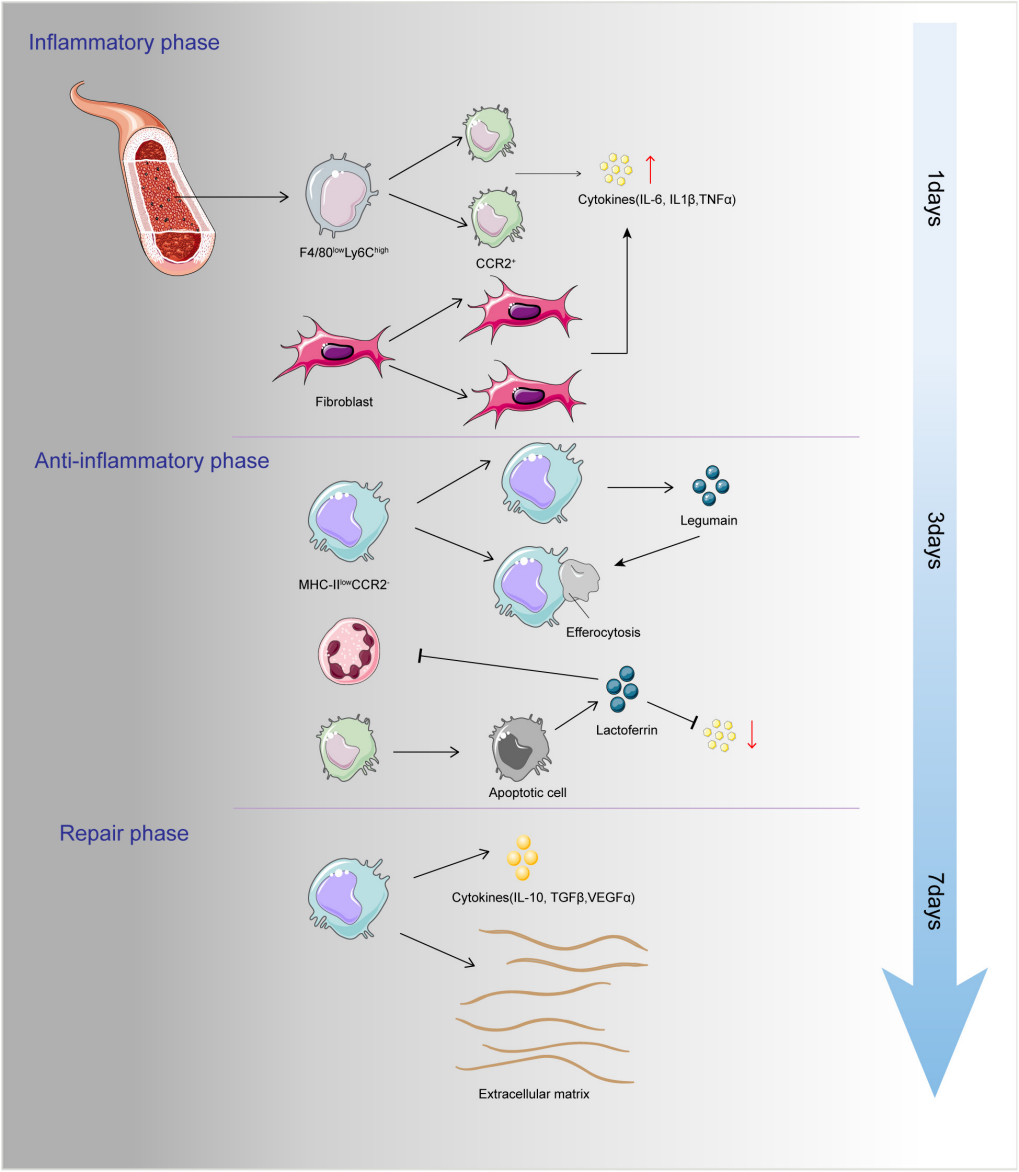
Macrophages dynamics in myocardial infarction. The mouse MI model progresses through three stages within a few short days: Inflammation, anti‐inflammatory and repair. Acute MI triggers significant inflammation, leading to extensive necrosis of cardiomyocytes in the infarct area. F4/80^low^Ly6C^high^ monocytes predominantly infiltrate, while CRMs and fibroblasts proliferate, releasing cytokines like IL‐6, IL‐1β and TNFα. By day 3, inflammation‐related gene expressions decrease, macrophages enhance phagocytosis and proliferation and pro‐inflammatory macrophages undergo apoptosis. MHC‐II^low^CCR2^−^ macrophages express legumain (Lgmn) during this stage. By day 7, macrophages secrete extracellular matrix proteins and cytokines like IL‐10, TGFβ and VEGFα, indicative of repair.

Myocardial infarction is the most prevalent form of cardiac injury, typically resulting from the blockage of coronary blood vessels, causing a deprivation of oxygen and essential nutrients. Subsequently, myocardial fibrosis and remodelling ensue, impacting the contractile and relaxation function of the myocardium, ultimately leading to the development of heart failure. In the current research, the mouse MI model is classified into three distinct phases over a span of 3–4 days—the inflammatory, anti‐inflammatory (sometimes known as the proliferative) and repair phases^43^ (Figure 3). Macrophages assume varying roles in different stages of MI.^44^ Acute myocardial infarction provokes a robust inflammatory response, contributing to significant necrosis of myocardial cells and macrophages in the infarcted region. Resident macrophages and fibroblasts within the tissue proliferate via TLR activation, thereby generating cytokines like IL‐1α and IL‐1β to modulate the inflammatory process. As previously mentioned, CCR2 plays a crucial role in distinguishing between embryonic and bone marrow origins. Specifically, CCR2^−^ derived from the embryonic yolk sac acts primarily as a preventive measure against excessive inflammation. Conversely, CCR2^+^ macrophages originating from the bone marrow are recognized as the primary drivers behind initial monocyte recruitment and the subsequent inflammatory response.

With increasing age, the proportion of CCR2^+^ to CCR2^−^ macrophages in the heart tends to rise. Studies indicate that a higher prevalence of CCR2^+^ macrophages in the heart correlates with a poorer prognosis after experiencing a myocardial infarction.^45^ Moreover, specific depletion of CCR2^+^ (CCR2‐DTR) has been found to facilitate the transformation of macrophages into smaller, less inflammatory phenotypes.^46^ Additionally, it has been observed that CCR2‐deficient mice demonstrate an intensification in cellular clearance following myocardial infarction, suggesting an enhanced ability to remove damaged cells from the affected area.^47^ During the acute inflammatory phase of myocardial infarction (MI), a substantial influx of CCR2^+^ monocytes occurs, accompanied by the infiltration of their derived CCR2^+^ macrophages. This leads to the replacement of the F4/80^high^Ly6C^low^ CRM population, which has also been observed in autoimmune myocarditis models.^48^ Within the first day following MI, there is a predominant infiltration of F4/80^low^ Ly6C^high^ monocytes,^49^ and macrophages express genes that contribute to the inflammatory response; By the third day, the expression of inflammation‐related genes is downregulated, while macrophages demonstrate enhanced phagocytic and proliferative capabilities. Pro‐inflammatory macrophages undergo apoptosis, and neutrophils release membrane‐associated protein A1 and lactoferrin, which contribute to the alleviation of inflammation,^50^ these findings suggest a transition of macrophages into a reparative state. Recent studies have identified a subset of MHC‐II^low^CCR2^−^ resident macrophages in the early stages of myocardial infarction. These macrophages exhibit a distinct and significant expression of legumain (Lgmn), a pivotal regulatory factor enabling macrophages to exert efferocytic function;^51^ By the seventh day, macrophages secrete extracellular matrix proteins and display reparative characteristics. On the second day following myocardial infarction (MI), there is a 60% decrease in the population of CRM in the infarcted area. However, between days 4 and 28, their numbers gradually increase through in situ proliferation.^10^ This experiment demonstrates the detrimental impact of depleting tissue‐resident macrophages on post‐MI tissue remodelling. It is worth noting that the depletion of resident macrophages did not result in a severe inflammatory response. Instead, the beneficial remodelling effects mediated by CCR2 relied, at least partly, on surface receptors MERTK^47^ and CD36.^52^ The clearance actions facilitated by MERTK and CD36 receptors can enhance the secretion of IL‐10, TGF‐β, VEGF‐A and VEGF‐C. This, in turn, promotes the release of pro‐repair factors by CCR2^−^, thus reducing the duration of the severe inflammatory response. Moreover, the extracellular matrix plays a crucial role in the repair of the infarcted zone. The temporal influence of CRMs post‐myocardial infarction (MI), as well as their quantitative role in the regulation of cardiac homeostasis and the modulation of acute functions during MI, warrants detailed exploration. A controversy persists regarding the potential of infiltrating monocytes to undergo differentiation into CRMs. Evidence to the contrary indicates that TIMD4 expression is sustained on resident Td+ macrophages up to 28 days subsequent to the infarction event, and it seems that the recruited macrophage cohort does not transition into TIMD4^+^ phenotype.^10^ On the contrary, data are emerging to suggest that monocytes possess the innate capacity to differentiate into CRMs. Specifically, following macrophage depletion mediated by clodronate‐liposome administration, there has been documentation of blood Ly6c^Hi^ monocytes, marked in vivo with fluorescent beads, trafficking into the myocardial tissue and differentiating into enduring populations of cardiac macrophages.^11^ Consequently, it appears that the obstruction of CCR2 or the strategic inhibition of pivotal inflammatory mediators that orchestrate monocyte recruitment could result in propitious therapeutic outcomes, given that the amplification of CRMslevels is conducive to the facilitation of cardiac recuperation subsequent to myocardial infarction (MI). Notwithstanding, the strategy of cMac transplantation fails to emerge as an optimal therapeutic modality. Research strides have been made with the innovative technique of fabricating cell membrane‐derived extracellular vesicles (mevs) from CRM, entailing the encapsulation of thymosin β4 (Tβ4) within nanoparticulate structures, thus leading to the genesis of Tβ4‐enriched, membrane‐modified extracellular vesicles (Tβ4‐mmev) tailored for myocardial repair applications. Evidence underscores that Tβ4‐mmev has a significant role in fostering myocardial cell proliferation and orchestrating endothelial cell migration. Clinically, MI mice models treated with Tβ4‐mmev exhibited a notable diminishment in myocardial fibrosis coupled with an enhancement in vascular density, delineating the therapeutic potential of this innovative approach.^53^ It warrants considerable attention that an unselective approach toward monocyte depletion could precipitate unfavourable ramifications. This strategy may interfere with the essential process of phagocytic removal of tissue detritus, attenuate the abundance and functional vigour of macrophages that emerge from monocytes in the protracted phase of injury resolution, collectively manifesting in outcomes that are counterproductive to the convalescence subsequent to myocardial infarction.

Gaseous signalling molecules, commonly referred to as gasotransmitters, are endogenous small gas molecules that engage in numerous intracellular signalling pathways and play an indispensable role in human metabolism and regulation. Over the past four decades, research has established that classical gasotransmitters, such as nitric oxide (NO), carbon monoxide (CO) and hydrogen sulfide (H₂S), exhibit significant cardioprotective effects. More recently, noble gases have also begun to show promise in cardioprotection.^54^ For instance, considerable evidence indicates that NO possesses antihypertensive, anti‐myocardial hypertrophy and renal protective properties.^55^ In cardiomyocytes (CMs), NO enhances the expression of soluble guanylate cyclase (sGC), thereby activating the cyclic GMP (cGMP)/protein kinase G (PKG) signalling pathway, which counteracts myocardial hypertrophy and remodelling.^56^ In vascular endothelial cells (VECs), NO mitigates endothelial cell aging by activating telomerase activity and scavenging reactive oxygen species (ROS). Additionally, NO promotes VEC proliferation and migration through the S‐nitrosylation of multiple target proteins and inhibits apoptosis.^57^ H₂S protects the myocardium by inhibiting the migration of CD11b^+^Gr1^+^ bone marrow cells from the spleen to the bloodstream and ischemic myocardium induced by myocardial ischemia.^58^ In CSE knockout mice, markers associated with pro‐inflammatory M1 macrophages (ILB, IL6, TNF‐α) in the myocardial ischemic region are significantly elevated compared to wild‐type mice, whereas markers for anti‐inflammatory M2 macrophages (IL‐10, CD163) are markedly reduced. In vitro studies show that H₂S facilitates the internalization of β1‐integrin on macrophages and further activates the SrcFAK/Pyk2‐Rac signalling pathway, thereby enhancing macrophage migration. Mechanistic investigations reveal that H₂S regulates the transformation of macrophages to the M2 phenotype by increasing mitochondrial biogenesis and promoting fatty acid oxidation/degradation.^59^ A recent comprehensive review has synthesized the cardioprotective mechanisms of various gaseous signalling molecules.^54^

### 5.2 CRMs and non‐ischemic cardiomyopathy

In addition to ischemic cardiomyopathy, several factors including hypertension, diabetes and aging contribute to the development of non‐ischemic cardiomyopathy, resulting in substantial cardiac remodelling. Hypertension, in particular, can elevate cardiac workload, leading to myocardial hypertrophy and eventual fibrosis. Extensive research has underscored the involvement of macrophages in the pathogenesis of hypertension, primarily through the promotion of NOS3 uncoupling and the activation of excessive ROS generation, ultimately culminating in vascular oxidative stress and hypertension. Epidemiological studies have revealed a significant pro‐inflammatory phenotype in monocytes from hypertensive patients, accompanied by a notable increase in the levels of inflammatory factors in the serum.^60^ In preclinical studies, researchers often employ the transverse aortic constriction (TAC)‐induced (TAC) model and angiotensin II (AngII) infusion models in mice to simulate increased cardiac workload. Consistent with the aforementioned description of myocardial infarction, there is a noteworthy surge in macrophage numbers 1 week post‐TAC.^61^ Early ablation of resident macrophages using macrophage colony‐stimulating factor 1 receptor (CD115)‐blocking antibodies does not elicit cardiac dysfunction during the initial week, characterized bynot significantly alter myocardial hypertrophy, ejection fraction, or shortening fraction induced by cardiac pressure overload. Although mψs exhibit a minor effect on hypertrophy prior to cardiac overload, post‐TAC surgery, the expression levels of pro‐inflammatory cytokine IL‐6, anti‐inflammatory cytokine IL‐10 and transforming growth factor Tgfβ1 are elevated in mψs cells, suggesting an overall immune activation. Furthermore, the depletion of Res mψs cells exacerbates cardiac fibrosis, impairs the angiogenic response within the heart and accelerates the progression of heart failure. Conversely, models utilizing CCR2 knockout (KO) mice to deplete recruited macrophages exhibit reduced fibrosis symptoms. This investigation also underscores the functional phenotypic divergence between resident macrophages and monocyte‐derived macrophages under conditions of pressure overload,^62^ early inhibition of macrophage infiltration achieved through CCR2 antagonist RS‐504393^62^ or antibody‐mediated depletion of Ly6C^hi^CCR2^+^ monocytes via MC21^62^ in the TAC model significantly inhibits macrophage infiltration, T cell proliferation and the development of compensatory left ventricular hypertrophy. Consequently, this intervention holds promise for mitigating adverse late‐stage left ventricular remodelling and diastolic dysfunction. The ablation of macrophages has been demonstrated to considerably attenuate the augmentation of arterial pressure resulting from sustained angiotensin II administration and to diminish the production of vascular reactive oxygen species (ROS).^63^ Recent studies elucidate that the demethylase ALKBH5 in macrophages modulates the m6A modification of IL11, thereby participating in the differentiation of circulating macrophages into myofibroblasts within the context of angiotensin II‐induced hypertension.^64^ Intriguingly, angiotensin II exhibits no influence on the fibrotic transformation of RCMs. Strategically targeting circulating macrophages and downregulating ALKBH5 can improve angiotensin II‐induced cardiac fibrosis and cardiac dysfunction.^64^ This comprehensive review elucidates the intricate details of the underlying mechanisms.^65^

### 5.3 CRMs and myocarditis

Myocarditis commonly arises from viral infections or subsequent immune responses, and its aetiology can be divided into infectious and non‐infectious factors. Infectious factors encompass bacteria, fungi, protozoa, parasites, spirochetes, rickettsiae and viruses. Non‐infectious factors encompass immune‐mediated diseases (allergens, alloantigens, autoantigens) and toxic agents (drugs, heavy metals, biotoxic substances, physical trauma, etc.). Macrophages exhibit diverse roles in myocarditis within various contexts. In the hearts of mice with viral myocarditis, significant infiltration of circulating monocytes occurs while the number of resident macrophages in the heart markedly decreases. Myocarditis triggered by the administration of immune checkpoint inhibitors for cancer therapy can also manifest with the accumulation of CCR2+ macrophages.^66^ Viral infections prompt macrophages to secrete various cytokines, such as TNF‐α and IL‐1β, leading to the formation of an inflammatory cytokine storm that directly damages the myocardium. In mice with Coxsackievirus‐induced myocarditis, using liposome‐encapsulated clodronate to deplete macrophages results in an increased viral burden but reduces myocardial necrosis and fibrosis. However, the precise function of embryonic‐derived cardiac macrophages in viral myocarditis remains uncertain. Interestingly, in autoimmune myocarditis, a similar approach yields contrasting results and improves cardiac function. Another study indicates that macrophages play distinct roles in the progression of myocarditis. In a murine model of experimental autoimmune myocarditis (EAM), two distinct subsets of monocytes, characterized as Ly6c^hi^ and Ly6c^lo^, are observed to infiltrate the cardiac tissue. During the acute phase of EAM, abundant IL‐17A weakens efferocytosis of Ly6clo monocyte‐derived macrophages (MDMs) by cardiac fibroblasts, thereby impeding their differentiation into macrophages. Conversely, in the absence of IL‐17A signalling, Ly6c^hi^ MDMs function as potent phagocytes with reduced pro‐inflammatory effects, while Ly6c^lo^ monocytes regain their differentiation into MHCII^+^ macrophages.^67^ Overstimulation of the myocardial sympathetic system is primarily governed by the active deployment and proliferation of embryonic‐origin resident macrophages, with only a nominal rallying of non‐classical Ly6C^lo^ monocytes.^68^ Despite the divergent functional profiles assumed by macrophages, they have emerged as strategic points of intervention in a spectrum of myocarditis models.^48^, ^69^ Despite the divergent functional profiles assumed by macrophages, they have emerged as strategic points of intervention in a spectrum of myocarditis models.^70^

Giant cell myocarditis (GCM) is a severe pathological condition characterized by extensive mixed inflammatory infiltrates predominantly composed of macrophages, accompanied by a substantial number of lymphocytes and multinucleated giant cells derived from macrophages, typically exhibiting a diffuse distribution. Eosinophils and plasma cells are less frequently expressed. The giant cells are CD68^+^ macrophages, usually located at the periphery of the inflammatory lesions.^71^ This type of myocardial inflammation primarily affects previously healthy young adults, with an unknown aetiology and is often associated with progressive heart failure, refractory ventricular arrhythmias and conduction system abnormalities, resulting in a high mortality rate. Up to 20% of GCM cases occur in patients with autoimmune diseases.^72^ In experimental mouse models, cyclosporine (an immunosuppressant that inhibits T cell activation signalling) and anti‐α/β T cell receptor antibodies have been shown to prevent the development of GCM.^73^ However, the detailed inflammatory response in GCM, particularly the formation of multinucleated giant cells, remains unclear. A recent study^74^ conducted single‐cell sequencing of Cd45^+^ cells from GCM rats, identifying immune cell types within multinucleated giant cells, excluding B cells but including CD4^+^ T cells, CD8^+^ T cells, neutrophils and giant cells. Neutrophils can recruit other immune cells, especially macrophages and T cells, through Ccdc25. Inhibition of NET formation can reduce immune cell infiltration in GCM. Macrophages are the primary source of giant cells, and the authors found that macrophages constitute the largest cell population in GCM, which can be classified into five categories, with Arg1^+^ and C1QA^+^ macrophages potentially involved in the pathogenesis of GCM.

### 5.4 CRMs and cardiac regeneration

Cardiac tissues in neonatal rodents are endowed with an intrinsic regenerative potential that is conspicuously absent in mature cardiac counterparts. Cumulative evidence underscores the contributory role of macrophages in mediating the process of cardiac regeneration. These immune effector cells exhibit the capabiltiy to secrete OSM, and within the context of cardiac regeneration in zebrafish—a species known for its regenerative capabilities—it has been discerned that macrophages can facilitate myocardial renewal independent of revascularization pathways. This is achieved through their specific recruitment within the epicardial‐myocardial niche, subsequently enhancing the transcription of Vegfa within the epicardial domain and reinforcing endothelial notch signalling, thereby amplifying the myocardial proliferative cascade.^75^ Notwithstanding, the intricate molecular and cellular underpinnings germane to the involvement of macrophages in cardiac regenerative phenomena, as well as the modalities through which macrophages galvanize the epicardium, remain to be comprehensively decoded. Illustrative of the therapeutic potential of these findings, recent rodent‐based investigations have showcased that transplantation of macrophages harvested from neonatal mice post‐apical resection into adults with experimental myocardial insults engenders substantive cardiac restitution and cellular proliferation,^76^ pending further elucidation of the underlying mechanistic intricacies. Employment of a clodronate liposome‐mediated approach to selectively deplete macrophages in the context of neonatal murine models of myocardial infarction has precipitated pronounced augmentation of fibrotic scarring, deterioration of myocardial performance and the stunting of neovascularization.^77^, ^78^ These manifestations succinctly denote the indispensability of macrophages within the cardiac microenvironment of neonatal mice, signifying their instrumental role in the promotion of cardiac tissue regeneration. The quintessential involvement of these immune cells is increasingly recognized as being central to the orchestration of effective regenerative responses post‐myocardial injury, highlighting their prospective value as targets for therapeutic intervention in cardiac recuperation.^79^ Another study corroborates the evidence,^80^ it delineates that subsequent to myocardial injury, there is a preferential expansion of embryonic origin MHC‐II^low^CCR2^−^ macrophages within the neonatal murine cardiac tissue. This expansion is concomitant with a moderated inflammatory response, which is characterized by slight upregulation of inflammatory mediators such as Mcp1, Mcp3, IL6 and IL1β. Conversely, mature cardiac tissue demonstrates a propensity to recruit MHC‐II^high^CCR2^+^ macrophages, which serves to amplify the inflammatory milieu. Examination of the conditioned medium derived from CCR2^−^ neonatal macrophages elucidated that the medium significantly facilitated endothelial tubulogenesis, which in turn, notably enhanced the proliferative response of neonatal rat cardiomyocytes. Conversely, the conditioned medium procured from CCR2^+^ adult macrophages did not induce endothelial tube formation within the confines of an in vitro environment, thus highlighting a potential intrinsic property of neonatal macrophages to expedite myocardial regeneration via the dual mechanisms of cardiomyocyte proliferation and angiogenesis. Considering the contributory role of macrophages in cardiac regeneration, it is postulated that the integration of endogenous cardiac macrophages into bioengineered myocardial tissue assemblies might more accurately mirror the physiological conditions inherent to the native myocardium. Prior in vitro investigations have demonstrated that macrophages sourced from peripheral blood possess the ability for cellular communication with cardiomyocytes derived from stem cells, mediated by the BMP signalling cascade.^81^ Recent studies have elucidated that biofabricated myocardial tissues harbour a heterogeneous cohort of macrophages, and they undergo phenotypic transfiguration concomitant with the processes of de‐differentiation and structural reconfiguration within these engineered cardiac constructs.^82^ An auspicious research area is the application of engineered heart tissue (EHT) in tandem with macrophages to facilitate the screening of anti‐inflammatory agents in the context of myocardial infarction therapies.^83^, ^84^ Despite the fact that a spectrum of anti‐inflammatory pharmacologic agents, including non‐steroidal anti‐inflammatory drugs, glucocorticoids and ciclosporin, have statistically been linked with enhanced survival rates, their capacity to deliver direct cardioprotective effects is often inadequate. The strategic employment of targeted anti‐inflammatory drugs presents a potential solution to these observed deficits. For instance, within the landmark CANTOS (Canakinumab Anti‐inflammatory Thrombosis Outcomes Study) cardiovascular trial, the therapeutic canakinumab was leveraged for its efficacy in the targeted suppression of inflammation via the inhibition of IL‐1β, which led to a downstream reduction in IL‐6 and high‐sensitivity C‐reactive protein (hsCRP) levels. Nonetheless, there exists an ongoing challenge wherein studies predominately utilize macrophages isolated from peripheral blood, a practice that raises concerns regarding their comparability and therapeutic parity with the native, tissue‐resident macrophages; this is attributed to the profound differences delineated in their respective transcriptomic and epigenomic profiles, as previously detailed.^85^ However, the extraction of tissue‐resident macrophages from essential organs remains largely untenable, a consequence of stringent ethical considerations coupled with technical constraints. In light of these limitations, investigators are increasingly optimistic about harnessing the potential of stem cells to catalyse the differentiation of progenitor macrophages. Over recent decades, a multitude of differentiation protocols have been meticulously established, which result in macrophages with a CD14^high^CD16^low^CD163^+^CD11b^+^ profile,^86^, ^87^, ^88^ closely mirroring the phenotype of macrophages that originate from peripheral blood. Despite these advances, the scientific community has yet to devise a reliable methodology for the induction of differentiation into macrophages that are native to the cardiac tissue. Nonetheless, a promising avenue has emerged through the utilization of co‐culture systems, which have proven effective in engendering tissue‐specific macrophages. Illustratively, macrophages that have been induced and then co‐cultured with neuronal cells have been shown to adopt attributes akin to microglial cells.^89^ In a parallel fashion, similarly induced macrophages, upon co‐culturing with hepatocytes, begin to demonstrate characteristics resembling those of Kupffer cells.^90^ This inquiry inevitably surfaces: could one orchestrate a co‐culture of induced macrophages with cardiomyocytes, or alternatively employ a culture medium that has been conditioned by cardiomyocytes, for the successful in vitro derivation of cardiac resident macrophages?

### 5.5 CRMs and cardiac aging

Cardiac aging is an independent risk factor for the high incidence and mortality rates of cardiovascular diseases, such as coronary artery disease and heart failure. Structural characteristics of cardiac aging include left ventricular hypertrophy, a decrease in the number of cardiomyocytes and fibroblast proliferation, leading to increased fibrosis.^91^ Studies in aging mice show a gradual increase in cardiac macrophage numbers after 18 months of age, with this increase being positively correlated with age.^92^, ^93^ During the aging process, the self‐renewal and proliferative capacities of CRMs decline, leading to a reduction in their numbers and proportion, while circulating CCR2^+^ macrophages gradually come to dominate the cardiac macrophage population.^45^

As the number of macrophages increases during aging, their capacity to secrete MMP‐9 and CCL2 is enhanced, along with the upregulation of pro‐inflammatory factors such as tumour necrosis factor (TNF‐α), interleukin‐6 (IL‐6), matrix metalloproteinases (MMPs) and cytokine ligands (CCL2). This upregulation is positively correlated with left ventricular enlargement, indicating that macrophages play a key role in the increased inflammatory response during aging.^94^ Additionally, aging is associated with immune system senescence, increasing susceptibility to cardiovascular diseases. Studies have shown that myocardial fibrosis in aged mice may result from age‐dependent inflammatory immune dysregulation. Normal aging cells can release cytokines and chemokines that attract macrophages to lesions, while aged macrophages exhibit reduced phagocytic function and decreased capacities to produce nitric oxide and hydrogen peroxide.^95^ Furthermore, their cell membrane expression of class II MHC molecules is reduced.^96^

Macrophages appear to have dual roles—they eliminate senescent cardiac parenchymal cells while also promoting aging through inflammatory processes. Cardiac macrophages maintain cardiac homeostasis by removing senescent or damaged cardiac cells. Factors such as MMP‐9 secreted by CRMs also increase with aging, which in turn promotes cardiomyocyte hypertrophy, extracellular matrix homeostasis disruption and collagen deposition, a hallmark of fibroblast senescence.^97^ However, the specific macrophages associated with cardiac aging and their relationship with acute or chronic inflammation remain unclear. Further single‐cell omics studies are needed to elucidate these relationships at the cellular level.

### 5.6 Exosomes in regulating the crosstalk between macrophages and cardiac cells

Exosomes are extracellular vesicles first discovered in 1983 from the reticulocytes of sheep. They can be secreted by various types of cells in the body and exert protective effects in ischemic myocardium through different mechanisms depending on their cellular origin. Recent studies have identified that exosomes from mesenchymal stem cells (MSCs),^98^, ^99^ plasma‐derived exosomes,^100^ cardiomyocyte‐derived exosomes,^101^ cardiac fibroblast‐derived exosomes,^102^ and macrophage‐derived exosomes^103^ can alleviate inflammation and myocardial injury by regulating macrophage polarization and migration. The promotion of macrophage polarization by exosomes from different cellular sources may be a key mechanism in their role in inflammatory modulation and myocardial infarction remodelling.

MSC‐derived exosomal miR‐486‐5p, for example, inhibits the expression of the phosphatase gene PTEN in hypoxic H9C2 cardiomyocytes, thereby activating the downstream PI3K/AKT pathway, which ultimately induces H9C2 cardiomyocyte proliferation and reduces apoptosis.^104^ Turner et al.^105^ compared exosomes derived from hiPSC‐CM from patients with left ventricular hypertrophy (LVH) and those from individuals with normal left ventricular mass. Co‐culturing these exosomes with hiPSC‐ECs resulted in significant expression changes related to angiogenesis and endothelial cell vascular formation. Exosomes from LVH patients significantly increased the proliferation of hiPSC‐ECs but decreased tube formation and migration, indicating angiogenesis dysregulation. This highlights the important role of exosomes as mediators of intercellular communication. Liu et al.^106^ found that several pro‐inflammatory miRNAs, including miR‐155, are highly expressed in M1‐exosomes (M1‐exos) following myocardial infarction. M1‐exosomes, which secrete high levels of miR‐155, can transfer from macrophages to cardiac fibroblasts, leading to increased incidence of cardiac rupture and exacerbated inflammation. They can also be transferred to endothelial cells, where they downregulate SIRT1/AMPKα2 and endothelial NO synthase, inhibiting endothelial cell angiogenesis. CPC‐derived exosomes (CPC‐exos) repair damaged myocardium and confer cardioprotective effects by regulating protein or target gene expression. High levels of miRNAs, including miR‐210, miR‐132 and miR‐146a‐3p, are enriched in exosomes secreted by CPCs. miR‐210 downregulates its known targets ephrinA3 and PTP1b, inhibiting cardiomyocyte apoptosis.^107^ miR‐132 downregulates its target RasGAP‐p120, enhancing tube formation in endothelial cells.^107^ Evidence suggests that CPC‐derived exosomes are more effective than BMC‐derived exosomes in preventing staurosporine‐induced cardiomyocyte apoptosis, possibly due to the presence of active forms of pregnancy‐associated plasma protein aPAPP‐A on the surface of CPC exosomes.^108^

As crucial messengers of intercellular communication, exosomes exert various cardioprotective effects through different signalling pathways. This positions exosomes as a promising new direction for myocardial infarction therapy. Studies have explored the use of monocyte/macrophage membrane vesicles as monocyte mimics and modified MSC exosomes to simulate monocyte inflammatory targeting characteristics. Monocyte‐derived exosomes (Mon‐Exos) accumulate significantly more in the heart than free exosomes, predominantly in the lesion areas of the left ventricular anterior wall. In mouse MI/RI models, Mon‐Exos have shown greater benefits in cardiac function and remodelling, endothelial cell maturation, neovascularization and immune modulation after treatment.^109^ Despite extensive research into the biological functions of exosomes, the mechanisms of exosome biogenesis, secretion and targeting to recipient cells remain incompletely understood and represent a future research direction.

## 6 THERAPEUTIC ROLE OF NLRP3 IN CARDIAC DISEASES

Chronic low‐grade inflammation is a significant risk factor for cardiovascular diseases. Members of the NOD‐like receptor (NLR) family are crucial in regulating inflammatory responses, with the NLRP3 inflammasome being the most representative and predominantly expressed in monocytes and macrophages. Research has demonstrated that in pressure‐overloaded cardiomyocytes, Ca^2+^/calmodulin‐dependent protein kinase II (CaMKII) and the subsequently activated NF‐κB can initiate the activation of the NLRP3 inflammasome, thereby leading to cardiac inflammation. Factors such as hyperglycemia, angiotensin II and ischemic cardiomyopathy can elevate reactive oxygen species (ROS), which are primary activators of NLRP3. The increased activity of the NLRP3 inflammasome following metabolic dysregulation correlates with heightened susceptibility to myocardial ischemic injury.^110^ Inhibition of NLRP3 has been shown to effectively delay the progression of myocardial fibrosis, suggesting that targeting NLRP3 could be a novel therapeutic approach for treating myocardial fibrosis.^111^, ^112^

Recent studies have shown that the P2Y12 receptor antagonist ticagrelor and the sodium‐glucose co‐transporter‐2 (SGLT2) inhibitor dapagliflozin can inhibit NLRP3 inflammasome activation, thereby mitigating the progression of diabetic cardiomyopathy and cardiac fibrosis in mouse models.^113^ Additionally, the CaSR inhibitor Calhex231 may reduce myocardial infarction‐related cardiac fibrosis by suppressing the macrophage autophagy‐NLRP3 inflammasome pathway.^113^ The drug pirfenidone, traditionally used for idiopathic pulmonary fibrosis, has recently been shown to alleviate myocardial inflammation by modulating the ROS‐NLRP3‐inflammasome‐mediated IL‐1β signalling pathway.^114^ Furthermore, NLRP3 plays a critical role in anthracycline‐induced cardiac fibrosis. For example, doxorubicin, a potent anticancer agent, is limited in clinical use due to its cardiac toxicity. Recent studies have found that NLRP3 inflammasome inhibitors can reduce interstitial myocardial fibrosis and preserve left ventricular systolic function in doxorubicin‐induced cardiomyopathy mouse models.^115^

Aortic stenosis, the most prevalent heart valve disease in the elderly, is primarily treated with transcatheter aortic valve replacement (TAVR). However, incomplete reverse remodelling of the left ventricle post‐TAVR is often characterized by persistent cardiac fibrosis, inflammation and hypertrophy. Recent research indicates that sacubitril/valsartan has cardioprotective effects after TAVR by reducing NLRP3 inflammasome activation.^116^

Thus, the NLRP3 inflammasome is closely associated with various cardiovascular diseases, and small molecule inhibitors targeting NLRP3 may offer significant therapeutic potential in clinical practice. Several small molecules, such as colchicine,^117^ CY‐09,^118^ and OLT1177,^119^ have been identified as NLRP3 inhibitors, demonstrating protective effects in cardiovascular diseases. However, their safety, stability and specificity require further investigation.

## 7 CONCLUSION

In this comprehensive review, we have explored the ontogeny, distribution and transcriptional diversity of CRMs, along with their roles in physiological and pathological contexts. The intricate regulatory mechanisms governing cardiac macrophages remain subjects of critical inquiry. For example, the processes involving the turnover or integration of macrophages from disparate origins within the cardiac milieu are exceedingly complex. Moreover, the inflammatory response engenders a paradoxical effect upon myocardial injury; adeptly modulating the inflammatory and reparative capabilities of resident macrophages at opportune junctures is imperative for the translation of their functions into therapeutic interventions. Notably, the bulk of CRMs research has been conducted using murine models, highlighting the interspecies variances that may exist between humans and rodents. Thus, achieving a profound understanding of macrophage functionalities within the spectrum of cardiovascular diseases necessitates the development of more representative animal models as well as the advancement of more sophisticated investigative modalities.

In conclusion, we have identified several critical future research questions concerning the role of macrophages in cardiovascular diseases. First, a comprehensive understanding of macrophage heterogeneity is essential for elucidating the role of macrophages in cardiovascular diseases. Therefore, it is imperative to further investigate macrophage heterogeneity and its intricate regulatory networks under pathological conditions. Second, the regulation of macrophage functions should be studied from multiple perspectives, including epigenetic modifications, signalling pathways, environmental factors and metabolic processes, from embryonic development to adulthood. Third, anti‐inflammatory therapy has emerged as a promising strategy for cardiovascular disease intervention, demonstrating significant potential. Future therapeutic approaches should focus on developing precision treatments based on macrophage heterogeneity.

## AUTHOR CONTRIBUTIONS

**Yingnan Liao:** Funding acquisition (lead); project administration (lead); software (supporting); writing – original draft (lead). **Liyuan Zhu:** Data curation (lead); visualization (equal); writing – original draft (equal).

## CONFLICT OF INTEREST STATEMENT

The authors have no conflicting financial interest.

## Data Availability

The data that support the findings of this study are available from the corresponding author upon reasonable request.
